# Transcriptome analysis of *Rafflesia cantleyi* flower stages reveals insights into the regulation of senescence

**DOI:** 10.1038/s41598-021-03028-x

**Published:** 2021-12-08

**Authors:** Nur-Atiqah Mohd-Elias, Khadijah Rosli, Halimah Alias, Mohd-Afiq-Aizat Juhari, Mohd-Faizal Abu-Bakar, Nurulhikma Md-Isa, Mohd-Noor Mat-Isa, Jumaat Haji-Adam, Hoe-Han Goh, Kiew-Lian Wan

**Affiliations:** 1 School of Biosciences and Biotechnology, Faculty of Science and Technology, Universiti Kebangsaan Malaysia, 43600 Bangi, Selangor Malaysia; 2 Malaysia Genome and Vaccine Institute, Jalan Bangi, 43000 Kajang, Selangor Malaysia; 3 School of Environmental and Natural Resource Sciences, Faculty of Science and Technology, Universiti Kebangsaan Malaysia, 43600 Bangi, Selangor Malaysia; 4 Fraser’s Hill Research Centre, Faculty of Science and Technology, Universiti Kebangsaan Malaysia, 43600 Bangi, Selangor Malaysia; 5 Department of Biological Sciences and Biotechnology, Faculty of Science and Technology, Universiti Kebangsaan Malaysia, 43600 Bangi, Selangor Malaysia; 6 Institute of Systems Biology, Universiti Kebangsaan Malaysia, 43600 Bangi, Selangor Malaysia

**Keywords:** Plant biotechnology, Plant development, Plant molecular biology, Data acquisition, Genome informatics, Sequence annotation

## Abstract

*Rafflesia* is a unique plant species existing as a single flower and produces the largest flower in the world. While *Rafflesia* buds take up to 21 months to develop, its flowers bloom and wither within about a week. In this study, transcriptome analysis was carried out to shed light on the molecular mechanism of senescence in *Rafflesia*. A total of 53.3 million high quality reads were obtained from two *Rafflesia cantleyi* flower developmental stages and assembled to generate 64,152 unigenes. Analysis of this dataset showed that 5,166 unigenes were differentially expressed, in which 1,073 unigenes were identified as genes involved in flower senescence. Results revealed that as the flowers progress to senescence, more genes related to flower senescence were significantly over-represented compared to those related to plant growth and development. Senescence of the *R. cantleyi* flower activates senescence-associated genes in the transcription activity (members of the transcription factor families MYB, bHLH, NAC, and WRKY), nutrient remobilization (autophagy-related protein and transporter genes), and redox regulation (*CATALASE*). Most of the senescence-related genes were found to be differentially regulated, perhaps for the fine-tuning of various responses in the senescing *R. cantleyi* flower. Additionally, pathway analysis showed the activation of genes such as *ETHYLENE RECEPTOR*, *ETHYLENE-INSENSITIVE 2, ETHYLENE-INSENSITIVE 3*, and *ETHYLENE-RESPONSIVE TRANSCRIPTION FACTOR*, indicating the possible involvement of the ethylene hormone response pathway in the regulation of *R. cantleyi* senescence. Our results provide a model of the molecular mechanism underlying *R. cantleyi* flower senescence, and contribute essential information towards further understanding the biology of the Rafflesiaceae family.

## Introduction

*Rafflesia* is a genus of holoparasitic angiosperm from the family Rafflesiaceae. Among its family members, *Rafflesia* is the most species-rich genus, comprising over 30 species with many new species being described in recent years^1–3^. *Rafflesia* is unique among other flowering plants due to its highly reduced vegetative structures, yet produces the largest flower in the world^4^. The largest flower recorded is *Rafflesia arnoldii* measuring up to 100 cm in diameter^5^ and the vegetative structure exists as endophyte made up of a uniseriate strand that transitions to flowering abruptly^4^. The floral structure comprised of congenitally fused petal whorl, which made up the diaphragm and five brick red perianth lobes, corresponding to sepals in most angiosperms^6^.

*Rafflesia* has a very long-life cycle that can take up to four years^7^, followed by a short blooming period of four to eight days^8,9^. *Rafflesia cantleyi* takes between six to 21 months for flower buds to mature and their blooms were recorded to last for only five to eight days^10^. During the blooming period, female *Chrysomya chani* carrion flies will be attracted to the flower, allowing pollination to happen^11^. Subsequently, the flower will start to die and early symptoms of death or aging (also known as senescence) in *Rafflesia* include withering and discoloration of perigone lobes from red to blackish-brown. Eventually, death can be seen when the entire flower decomposes.

Senescence is the last developmental stage of a plant’s life cycle, which involves highly coordinated structural, biochemical, and molecular changes that lead to the death of organs and the whole plant^12^. Senescence involves death at the cellular level known as programmed cell death (PCD)^13^ and is known to be triggered by various environmental factors such as heat and light stresses, insect and pathogen attacks, wounding, and salinity^14^. Studies of flower senescence at the molecular level have revealed genes involved in various cellular activities, including protein degradation (e.g., *PRT22* in *Sandersonia*; *RbCP1* in *Rosa*), nutrient remobilization (e.g., *pDcCP1* in *Dianthus*; *DAFSAG2* in *Narcissus*), and nucleic acid degradation (e.g., *DSA6* in *Hemerocallis*; *PhNUC1* in *Petunia*; *DcNUC1* in *Dianthus*)^15–17^. Other than that, genes related to various phytohormone pathways such as ethylene biosynthesis (e.g., *1-AMINOCYCLOPROPANE-1-CARBOXYLATE (ACC) SYNTHASE (ACS)* and *ACC OXIDASE (ACO)* in *Dianthus*), ethylene induction (e.g., ethylene receptors *ETR1*, *ETR2*, *ERS1*, and *ERS2* in *Arabidopsis*; *DcERS1*, *DcERS2*, and *DcETR1* in *Dianthus*) and ethylene signaling (e.g., *CONSTITUTIVE TRIPLE RESPONSE 1 (CTR1)* in *Arabidopsis*; *RhCTR1* and *RhCTR2* in *Rosa*) are also known to be regulated by flower senescence^17^. As in other plants, senescence in *Rafflesia* is an important event. This is more so as the plant exists almost entirely as a single flower, and as such, senescence of the single organ correlates directly with plant death.

Although the uniqueness of *Rafflesia* has always been a fascination, the biology of this plant remains poorly understood. Part of this is due to sampling difficulties and hence a lack of molecular studies on this species. Previously, we conducted a transcriptomic study on the *R. cantleyi* flower^18^ with more comprehensive transcriptome analyses involving multiple stages of *R. cantleyi* floral buds^19,20^. Parts of these data were instrumental towards the discovery of its ability to retain some common plastid-associated processes, albeit missing the photosynthetic components^21^. In this study, the next-generation sequencing technology was used as a tool to generate transcriptome data of two different flower stages of *R. cantleyi*, which are defined by the approximate duration from anthesis, i.e., one day (flower stage 1; F1) and three days (flower stage 2; F2) after full flower blooming. The transcriptome was analyzed to identify novel senescence-associated genes and their regulation in *Rafflesia* flower senescence. The expression levels of selected genes was validated using reverse transcription-quantitative polymerase chain reaction (RT-qPCR).

## Results

### Transcriptome assembly and functional annotation

**Figure 1:**
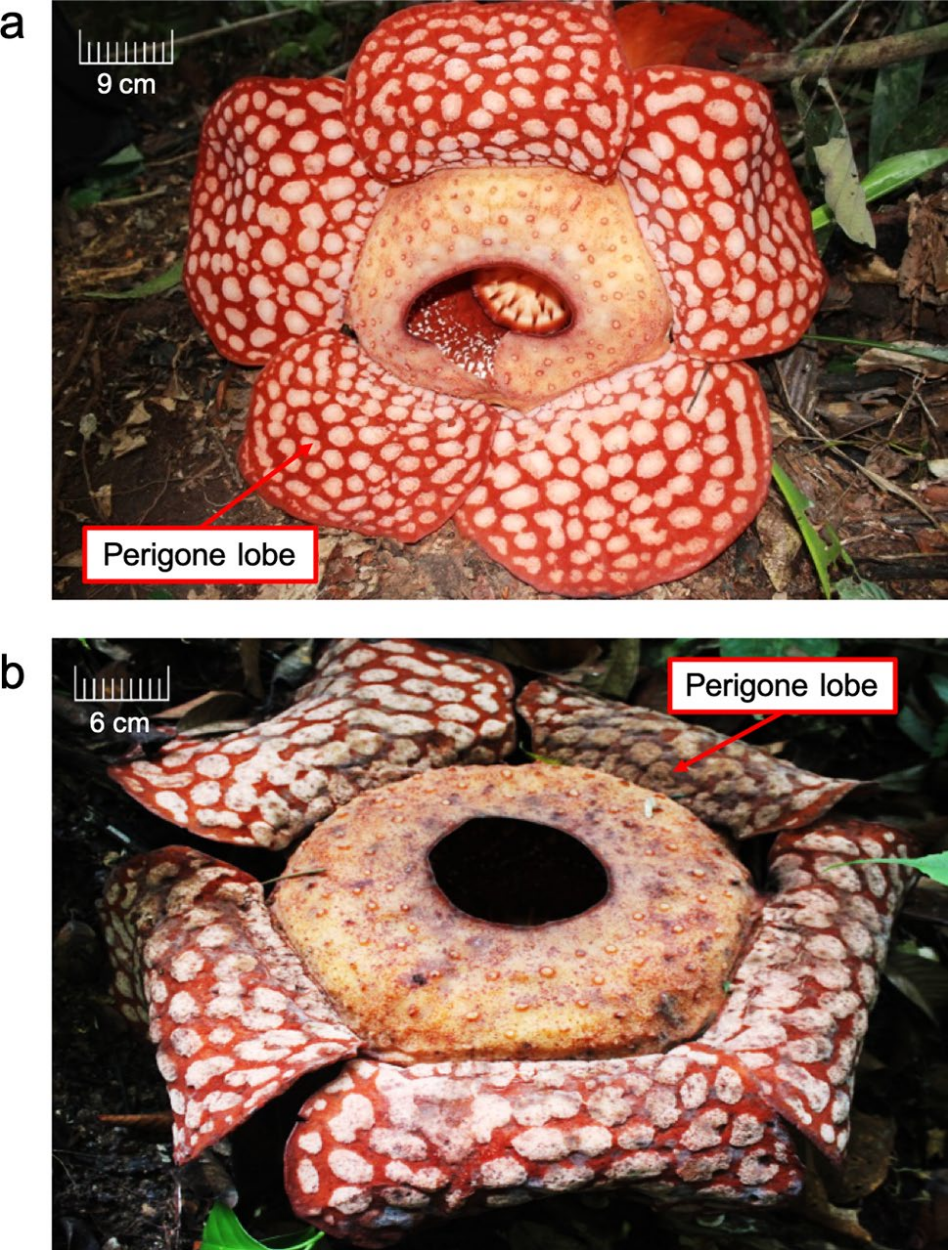
The *Rafflesia cantleyi* flowers. *R. cantleyi* samples (**a**) one day (F1) and (**b**) three days (F2) after blooming were collected from Bukit Lahar, Pahang, Malaysia. Tissue samples from the perigone lobes of the two different flowers were obtained for transcriptome sequencing.

Transcriptome sequencing was carried out on the perigone lobe of the F1 and F2 flower stages of the *R. cantleyi* flower (Fig. 1). *Rafflesia* flowers have a short blooming period of up to seven days before they wither and die. From our observation in the field (data not shown), flowers after three days of blooming showed significant fungal infections, which will affect endogenous gene expression. Thus, to minimize this effect on the transcriptome analysis, we chose to focus on the F1 and F2 flower stages in this study.

**Figure 2:**
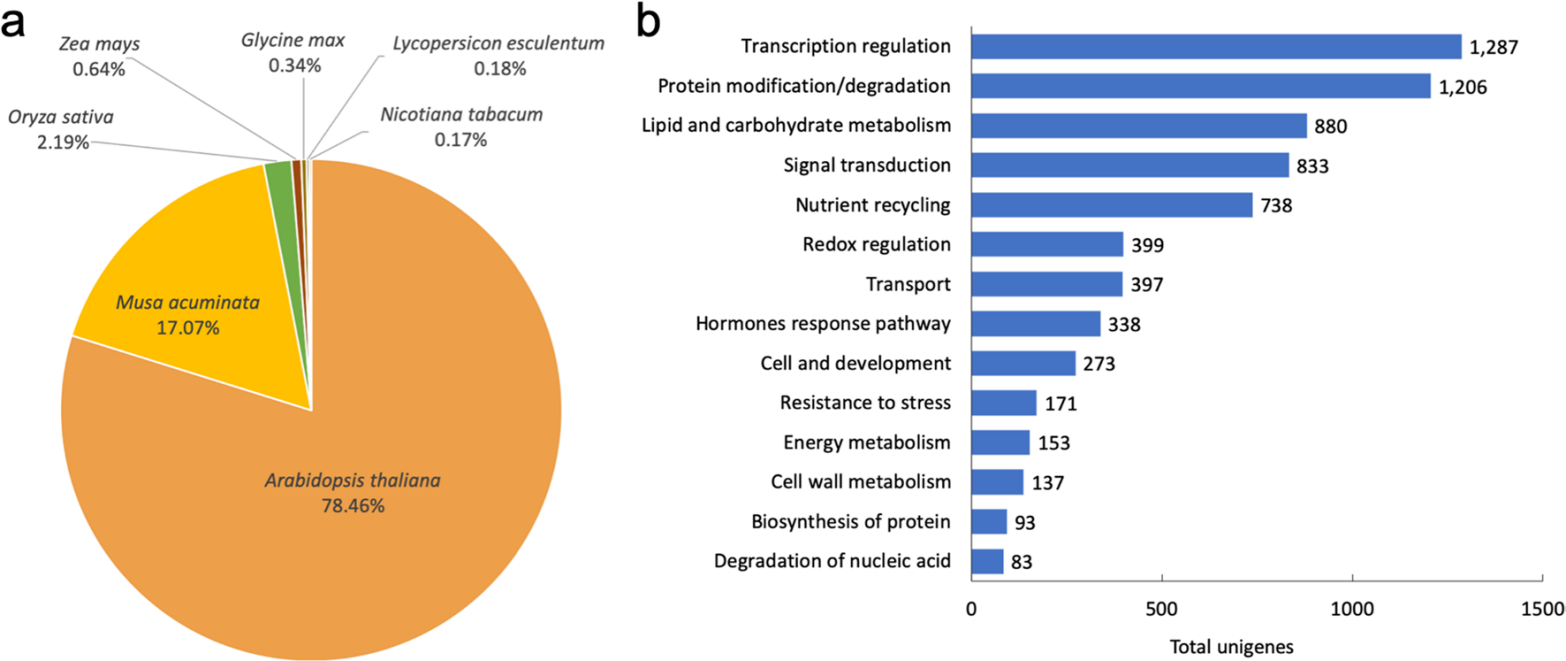
Annotations of *Rafflesia cantleyi* transcriptome data. (**a**) Species distribution of annotated *Rafflesia cantleyi* flower unigenes based on LSD 2.0. (**b**) Functional categories of annotated *Rafflesia cantleyi* flower unigenes based on LSD 2.0.

A total of 53.3 million cleaned reads from F1 and F2 were assembled using Trinity and iAssembler, generating 64,152 unigenes with an average length of 843 bp. BLASTX search resulted in a total of 39,234 (61%) and 26,775 (42%) unigenes matched to NCBI non-redundant (NR) and Swiss-Prot sequences, respectively. The similarity search performed on the unigenes against the Leaf Senescence Database (LSD 2.0) successfully identified 13,679 (21.3%) unigenes with significant matches to senescence-associated protein sequences (Supplementary Dataset S1). The BLAST analysis against LSD 2.0 (Fig. 2a) revealed that the majority of significant hits matches *Arabidopsis thaliana* (78.46%) and *Musa acuminata* (17.07%), followed by other plant species, such as *Oryza sativa* (2.19%), *Zea mays* (0.64%), and *Glycine max* (0.34%). Further classification of the unigenes into different categories according to their functions in senescence showed that the *R. cantleyi* unigenes are mostly involved in transcription regulation (1,287 unigenes), protein modification and degradation (1,206 unigenes), lipid and carbohydrate metabolism (880 unigenes), signal transduction (833 unigenes), and nutrient recycling (738 unigenes) (Fig. 2b).

### Differential expression analysis and GO enrichment

Differential expression analysis was conducted to identify unigenes with significant differential expression between F1 and F2. The results of this analysis showed that a total of 5,166 unigenes identified were differentially expressed between F1 and F2. Of these, 4,062 unigenes were significantly up-regulated (log_2_FC ≥ 2; FDR ≤ 0.05) and 1,104 unigenes were significantly down-regulated (log_2_FC ≤ -2; FDR ≤ 0.05). Further analysis from LSD 2.0 showed that 1,073 unigenes were identified to be involved in senescence, of which 715 unigenes were up-regulated and 358 unigenes were down-regulated (Supplementary Dataset S2).

**Figure 3:**
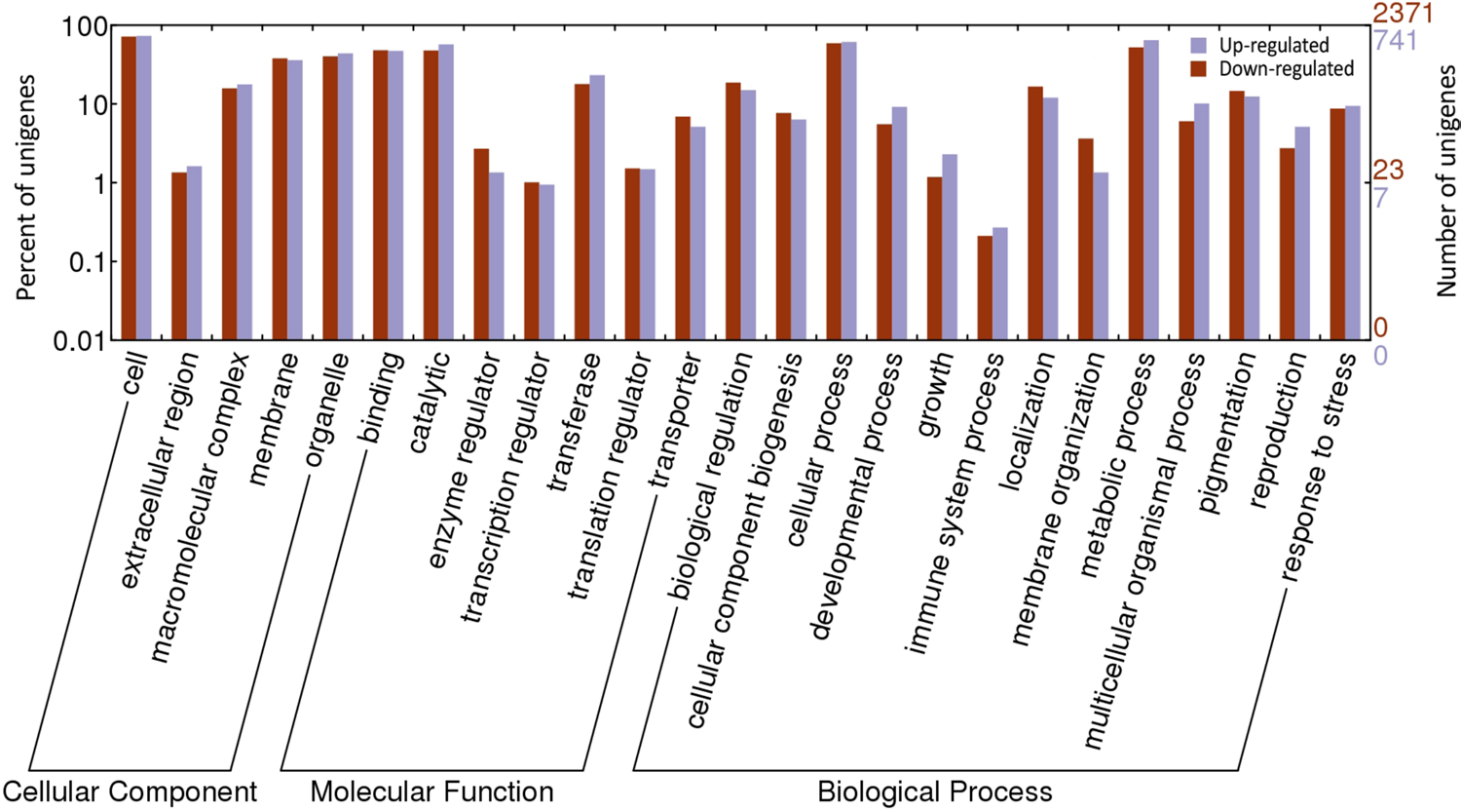
WEGO annotation plot of DEGs in *Rafflesia cantleyi*. The percentage and number of unigenes of each GO terms are indicated by the left and right x-axis, respectively. GO categories are indicated by the y-axis.

Gene ontology (GO) annotation carried out on the differentially expressed genes (DEGs) showed the highest match to cell (GO:0005623) and organelle (GO:0043226) for the cellular component category; catalytic activity (GO:0003824) and binding (GO:0005488) for the molecular function category; while the biological process category was dominated by metabolic (GO:0008152) and cellular process (GO:0009987) (Fig. 3). GO enrichment analysis of the DEGs was carried out to identify significantly over-represented DEGs (Supplementary Dataset S3). For up-regulated DEGs, 22 GO terms were significantly enriched, including transportation, lipid metabolism, cell cycle, response to stress, cellular protein modification process, nucleotide and protein binding, and enzyme regulator activity. In contrast, 27 GO terms for the down-regulated DEGs were significantly enriched, including meiotic nuclear division, response to biotic stimulus, post-embryonic development, and cell growth.

**Figure 4:**
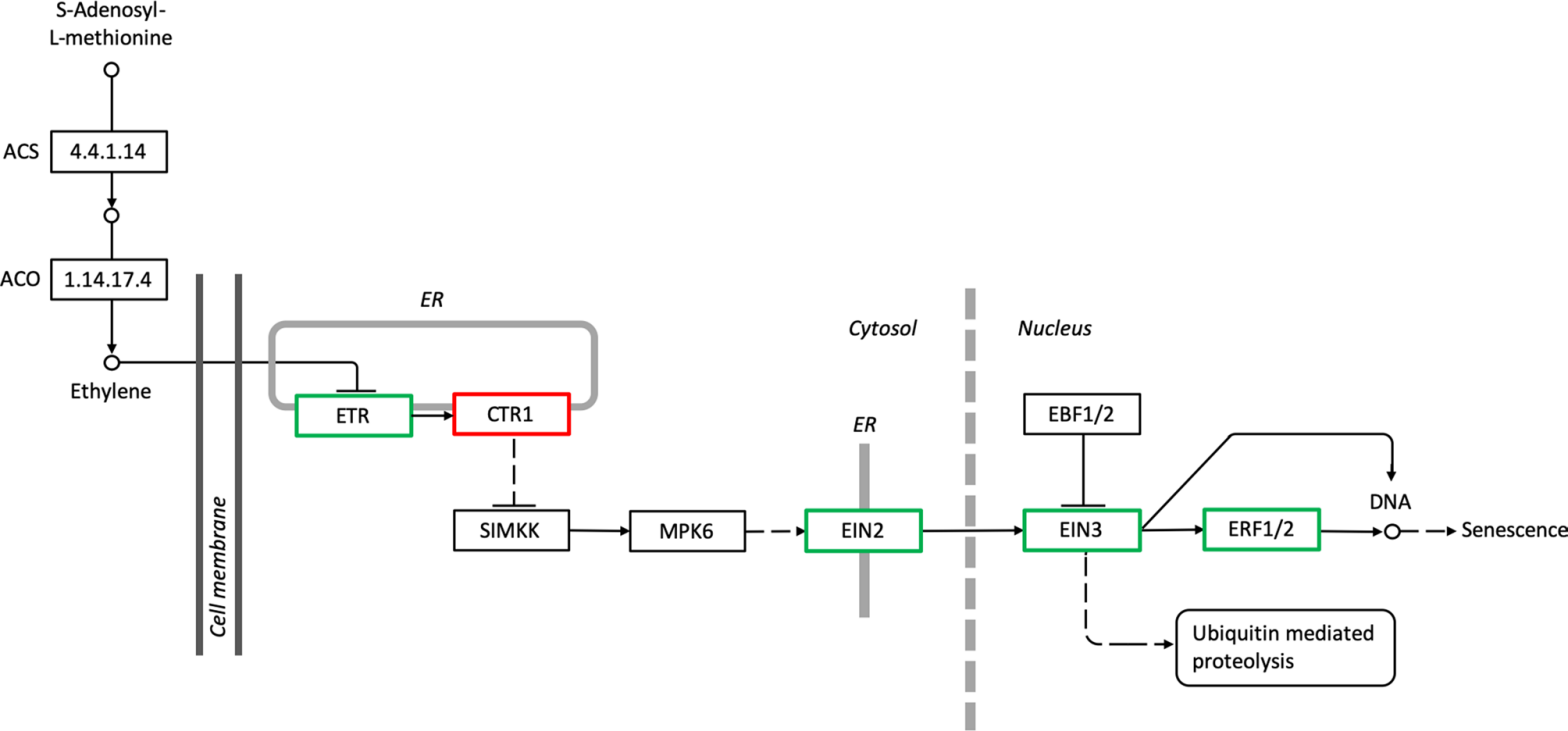
KEGG pathway of ethylene biosynthesis and signal transduction pathway mapped with *Rafflesia cantleyi* unigenes. Green outlined boxes represent up-regulated unigenes while the red outlined box represents a down-regulated unigene. *ER* endoplasmic reticulum; *ACO 1-AMINOCYCLOPROPANE-1-CARBOXYLATE OXIDASE*; *ACS 1-AMINOCYCLOPROPANE-1-CARBOXYLATE SYNTHASE*; *CTR1 CONSTITUTIVE TRIPLE RESPONSE 1*; *EBF1/2 EIN3-BINDING F-BOX PROTEIN*; *EIN2 ETHYLENE-INSENSITIVE 2*; *EIN3 ETHYLENE-INSENSITIVE 3*; *ERF1/2 ETHYLENE-RESPONSIVE TRANSCRIPTION FACTOR 1/2*; *ETR ETHYLENE RECEPTOR*; *MPK6 MITOGEN-ACTIVATED PROTEIN KINASE 6*; *SIMKK MITOGEN-ACTIVATED PROTEIN KINASE KINASE*.

Other than that, the DEGs identified showed functions in the regulation of transcription factors, nutrient remobilization, redox regulation, and ethylene regulation (Table 1; Supplementary Dataset S4). Members of the transcription factor families MYB, bHLH, NAC, WRKY, bZIP, MADS-box, and AUX/IAA were found to be differentially expressed between the two flower stages. Furthermore, the expression of autophagy-related genes (*ATG*) and transporter genes involved in nutrient remobilization were up-regulated. A slight difference in the expression pattern was recorded for unigenes involved in redox regulation, in which most genes were constitutively expressed at both stages. This pattern of constitutive expression was similar to unigenes involved in the ethylene biosynthesis but unigenes involved in the ethylene signal transduction showed significant differential expression (Fig. 4).

**Table 1:** Genes involved in senescence-related processes in *Rafflesia cantleyi* flower transcriptome and their functional annotation based on matches with the LSD 2.0 database.

Unigenes	Protein description	*Arabidopsis* gene IDs	Effects on senescence
**Transcription factor regulation**			
UN005301	bZIP transcription factor	AT1G75390	Unclear
UN021161	MADS-box-AGL8 protein	AT5G60910	Promote
UN010557	MYB7 protein	AT2G16720	Unclear
UN060295	WRKY transcription factor	AT4G31550	Unclear
UN019664	NAC transcription factor	AT5G13180	Delay
UN000182	Jumonji (JMJ) protein	AT5G46910	Unclear
UN027279	bHLH transcription factor	AT4G09820	Unclear
UN015470	RING/U-box family protein	AT5G41350	Unclear
UN016492	AUX/IAA transcription factor	AT3G23050	Unclear
UN017419	Homeobox protein	AT2G35940	Unclear
UN021998	C3H zinc-finger domain	AT5G58620	Unclear
UN005311	C2H2 zinc-finger domain	AT2G28200	Unclear
**Nutrient remobilization**			
UN020537	ATG4	AT2G44140	Delay
UN046014	ATG6	AT3G61710	Delay
UN027071	ATG7	AT5G45900	Delay
UN051413	ATG8	AT4G21980	Delay
UN049803	ATG9	AT2G31260	Delay
UN028827	Target of rapamycin (TOR)	AT1G50030	Promote
UN013404	ABC transporter	AT5G06530	Unclear
UN004449	Sugar transporter 14	AT5G26340	Unclear
UN026074	Nitrate transporter	AT1G32450	Unclear
UN015723	Polyamine transporter	AT1G31830	Unclear
**Redox regulation**			
UN004263	Catalase (CAT)	AT1G20630	Unclear
UN012078	Superoxide dismutase (SOD)	AT3G56350	Unclear
UN037470	L-Ascorbate oxidase (ASO)	AT1G76160	Unclear
UN001999	Peroxidase A2	AT5G06720	Unclear
UN063210	Peroxidase 17	AT2G22420	Unclear
**Ethylene pathway**			
UN007860	EIN2	AT5G03280	Promote
UN012030	ERF	AT3G23240	Unclear
UN025327	EIN3	AT2G25490	Delay
UN004001	CTR1	AT5G03730	Unclear
UN024806	ETR	AT1G66340	Delay
UN064036	ACO	AT1G05010	Unclear
UN023149	ACS	AT4G11280	Unclear

### Expression analysis of selected DEGs by RT-qPCR

**Figure 5:**
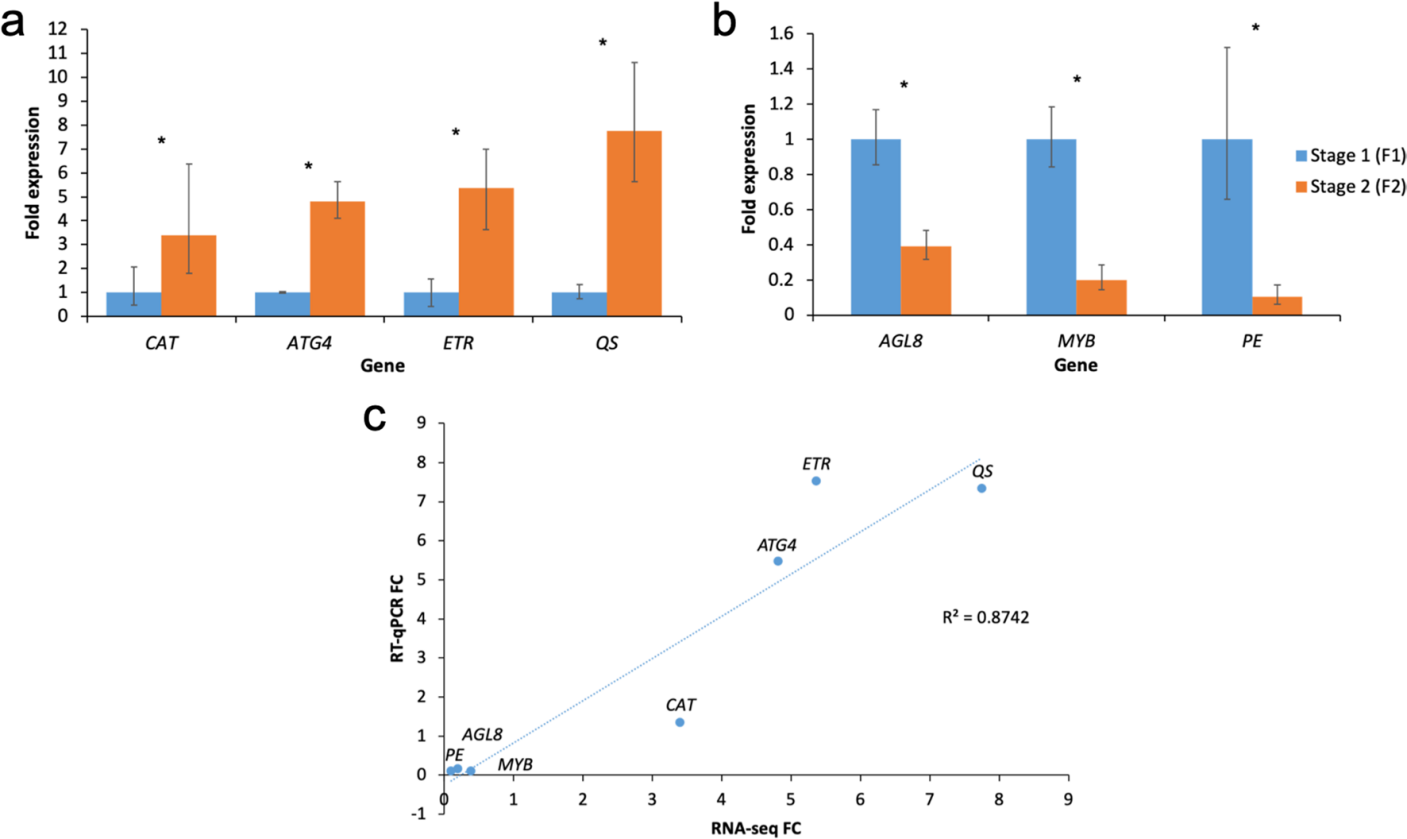
RT-qPCR analysis on the fold change of expression level at F2 relative to expression level at F1. (**a**) Unigenes with up-regulated expression. (**b**) Unigenes with down-regulated expression. (**c**) Correlation plot between the RT-qPCR fold-change (FC) compared to FC calculated from TPM values of RNA-seq analysis. Genes studied are *CAT CATALASE*, *ATG4 AUTOPHAGY-RELATED*, *ETR ETHYLENE RECEPTOR*, *QS QUINOLINATE SYNTHASE*, *AGL8 AGAMOUS-LIKE 8*, *MYB MYELOBLASTOSIS*, and *PE PECTIN ESTERASE*. Asterisks indicate significant differences (*P* < 0.05) in gene expressions.

Seven genes with different expression patterns between the two flower stages were selected for RT-qPCR validation analysis. Results showed that *QUINOLINATE SYNTHASE* (*QS*) has the highest increase in expression, followed by *ETHYLENE RECEPTOR* (*ETR*), *ATG4*, and *CATALASE* (*CAT*) (Fig. 5a), while *PECTIN ESTERASE* (*PE*) has the highest decrease in expression, followed by *MYB* and *AGL8* (Fig. 5b). Overall, the RT-qPCR results showed a high correlation with that of RNA-seq (R^2^ > 0.8742) (Fig. 5c).

## Discussion

In this study, besides the more commonly used databases such as NR and Swiss-Prot, a BLAST search was also performed against the leaf senescence-specific LSD 2.0 database. In the absence of a specific database for flower senescence, the LSD 2.0 database was utilized since most of the leaf senescence genes can also be found in the flower. Furthermore, leaf and floral organs arise from the same primordium, thus it is expected that the senescence genes are conserved in these two organs, as observed in *A. thaliana*^22^ and *Erysimum linifolium*^23^. BLAST results showed that the majority of the homologs are from *A. thaliana* (79.14%) and *M. acuminata* (16.98%), and this may be partly due to both of these species having the largest datasets in the LSD 2.0 database, 69.89% for *A. thaliana* and 16.46% for *M. acuminata*. GO annotation revealed that DEGs were mainly responsible for fundamental biological regulation and metabolism common in other plants. This suggests that *R. cantleyi* flower senescence is a coordinated biological process that could be regulated similarly to other plants^24^.

The transcriptional control mechanism, which involves various transcription factors, plays a pivotal role in regulating gene expression during plant development and senescence^25^. Many transcription factors have been identified previously and found to be differentially expressed during the development and senescence of various flower systems^17^. In *R. cantleyi*, members of MYB, bHLH, NAC, and WRKY were found to be up-regulated at F2. All of these families were known to be involved in various kinds of biological processes, including responses to stresses and injuries^26–29^. MYB102 functions in response to biotic and abiotic stresses, such as defense against insects, injuries, and osmotic stresses^30–32^, whereas, MYB7 is a transcription factor involved in the flavonoid biosynthesis induced during salinity stress^33^. On the other hand, the function of bHLH family members MYC2^34^, bHLH092^27^, and ICE1^35–37^ in abiotic stress response has been well characterized. Moreover, expression data from genome-wide transcriptome analyses in many plants such as *A. thaliana*^38^, soybean^39^, rice^40^, and *Chrysanthemum lavandulifolium*^41^ revealed that a significant proportion of NAC genes are related to stress responses^42^. Studies conducted on the expression of WRKY showed rapid induction when plants are exposed to a variety of stresses or defensive signals, including plant defense against attack and senescence^29,43,44^.

The up-regulation of these transcription factors is in line with other studies on flower senescence. The same phenomenon can be seen in *Dianthus* and *Gardenia*, which showed a high level of expression in MYB during flower senescence^45,46^. Other than that, the expression pattern of bHLH between the two *R. cantleyi* flower stages is similar to that in *Gardenia*, whereby this transcription factor family plays a major role in regulating flower senescence^46,47^. The expression pattern of NAC in the *R. cantleyi* flower was similar to that of *A. thaliana* leaf and flower senescence^22,48^.

The transcription factors that showed significant down-regulation in *R. cantleyi* were identified to be from the bZIP, MADS-box, and AUX/IAA families, which play important roles in flower development^49–51^. The transcription factors from the bZIP family plays important roles in regulating seed maturation and flower development^49^. These functions are also shared with the MADS-box transcription factors, which are known to be involved in the development of the reproductive structure and determination of meristems and organ identity^50^. Additionally, AUX/IAA transcription factors are also involved in many aspects of plant development. IAA1, IAA3, and IAA4 were identified to play key roles in development, particularly in root morphology and gravitropism, stem elongation, and inflorescence formation in *A. thaliana*, *O. sativa*, and *Eucalyptus*^51–53^. The down-regulation of these transcription factors suggests their reduced function during flower senescence. Overall, transcription factors involved in various responses to stresses, injuries, and senescence were up-regulated contrary to the down-regulation of transcription factors involved in flower development.

Flowers have a fixed lifespan determined by their status in sexual reproduction such that flowers that have been pollinated or are no longer receptive to pollination will be programmed to senensce^54^. In some plant species, the symptoms of flower senescence are visibly shown through wilting or withering of petals, whereas in other plants, the petals abscise while still turgid^55^. For plant species with petal wilting, such as in *Rafflesia*, the main function of flower senescence is proposed to allow the remobilization of nutrients to other parts of the plant^16,56,57^. One of the processes during the remobilization of nutrients is protein degradation, which involves multiple protease enzymes. These enzymes degrade proteins by hydrolyzing internal peptide bonds, or more commonly known as proteolysis, and this activity is irreversible.

Cysteine protease is an enzyme that has been reported to be involved in the remobilization of essential nutrients from senescing floral tissue. In some plant species such as *Sandersonia*^58^, *Narcissus*^59^, *Alstroemeria*^56^, and *Rosa hybrida*^60^, genes encoding cysteine protease were found to be induced during flower senescence. In this study, a unigene encoding cysteine protease in the *R. cantleyi* flower was identified. Differential expression analysis showed that the cysteine protease gene *ATG4* (UN020537) was up-regulated at F2 (Table 1; Supplementary Dataset S4). This gene was identified to be involved in autophagy, which is the main pathway for the degradation of proteins in vacuole activated by environmental stresses^61^.

Other than that, unigenes encoding other related autophagy proteins were also identified. Besides *ATG4*, the up-regulation of *ATG7*, *ATG8*, and *ATG9* at F2 were also similar to those in *A. thaliana* during leaf senescence^62^. The expression patterns of *ATG4* and *ATG8* in *R. cantleyi* are similar to *Ipomea nil* and *Petunia* with increasing expression during petal senescence^63–65^. Furthermore, the activation of the autophagy pathway is negatively regulated by *TARGET OF RAPAMYCIN* (*TOR*). The down-regulation of *TOR* caused the autophagy pathway activation in *A. thaliana*^66^. In this study, a unigene that encodes *TOR* was down-regulated at F2, indicating that the autophagy mechanism in the *R. cantleyi* flower is probably activated by the regulation of *TOR*.

Besides that, unigenes involved in transporter activity, such as ABC transporter and transporters of sugar, nitrates, and polyamines were also identified as DEGs (Table 1; Supplementary Dataset S4). In *A. thaliana*, the expression of transporter protein was found to be up-regulated during leaf senescence, which reflects nutrient remobilization during senescence^67^. Overall, the differential expression of unigenes related to nutrient remobilization suggests transportation of nutrients from the senescing flower of *R. cantleyi*, perhaps for the development of seeds and fruits.

The reactive oxygen species (ROS) represent free radicals derived from oxygen molecules, such as singlet oxygen (^1^O_2_), superoxide (O_2_·^−^), hydroxyl (OH·), hydroperoxyl (HO_2_·^−^), and hydrogen peroxide (H_2_O_2_)^68^. These radicals can oxidize various molecules in plants, which gives adverse effects on membrane integrity and protein stability leading to programmed cell death^69^. ROS are produced during normal plant development and the production increases when triggered by environmental stresses such as drought, salinity, and pathogen attack. ROS are extremely harmful in high concentrations, thus they need to be removed from the cell through various activities of antioxidative enzymes, such as superoxide dismutase (SOD), catalase (CAT), and ascorbate oxidase (ASO). In previous studies of flower senescence, such as in *Gladious*^70^, *Hemerocallis*^71,72^, and *Rosa hybrida*^73^, various free oxygen radicals were detected in flower petals. The increase of these free radicals caused the up-regulation of antioxidative enzymes SOD, CAT, and ASO during flower senescence^55^. In this study, unigenes that are involved in redox regulation encoding antioxidative enzymes CAT (UN004263), SOD (UN012078), ASO (UN037470), and other peroxidases (UN001999, UN063210) were identified (Table 1; Supplementary Dataset S4). Our results revealed that these unigenes showed constitutive expressions at both flower stages, except *CAT* (UN004263), which was found to be up-regulated in F2. High expression of ROS-related unigenes at both flower stages indicates the regulation of redox activity in *R. cantleyi*.

Flower senescence involves highly coordinated events that are regulated by endogenous signals, such as phytohormones and environmental factors, including biotic and abiotic stresses^74^. Several plant hormones have been reported to be involved in senescence, of which ethylene and abscisic acid (ABA) function to induce flower senescence, while cytokinin, gibberellic acid (GA), and auxin delay the process^16,75^. In most plant species, ethylene is a phytohormone that initiates flower senescence^76^. Results from differential expression analysis were able to help identify genes involved in ethylene hormone regulation in *R. cantleyi.*

Ethylene is synthesized through cysteine and methionine metabolism pathways mediated by enzyme precursors ACO and ACS^77^. In this study, unigenes encoding ACO (UN064036) and ACS (UN023149) were constitutively expressed at both flower stages. However, unigenes involved in the ethylene signal transduction pathway were identified to be differentially expressed. Among them, positive regulators *ETHYLENE-INSENSITIVE 2* (*EIN2*) (UN007860), *ERF* (UN012030), and *ETR* (UN024806), and transcription factor *ETHYLENE-INSENSITIVE 3* (*EIN3*) (UN025327) were significantly up-regulated, whereas a negative regulator *CTR1* (UN004001) was significantly down-regulated at F2 (Table 1; Supplementary Dataset S4). ETR serves as a receptor that binds to ethylene gases and the loss of ETR function delays senescence in flowers and leaves as recorded in *Petunia* and *Nicotiana sylvestris*^78–80^. On the other hand, in the ethylene signal transduction pathway, the binding of ethylene to the ETR receptor causes the CTR gene to be inactive. The deactivation of CTR activates other downstream components such as transcription factors EIN2, EIN3, and ERF, followed by ethylene response^17^. Constitutive expression of unigenes involved in the ethylene biosynthesis and the up-regulation of unigenes involved in the ethylene signal transduction pathway suggest the role of ethylene regulation in flower senescence of *R. cantleyi* (Fig. 4).

**Figure 6:**
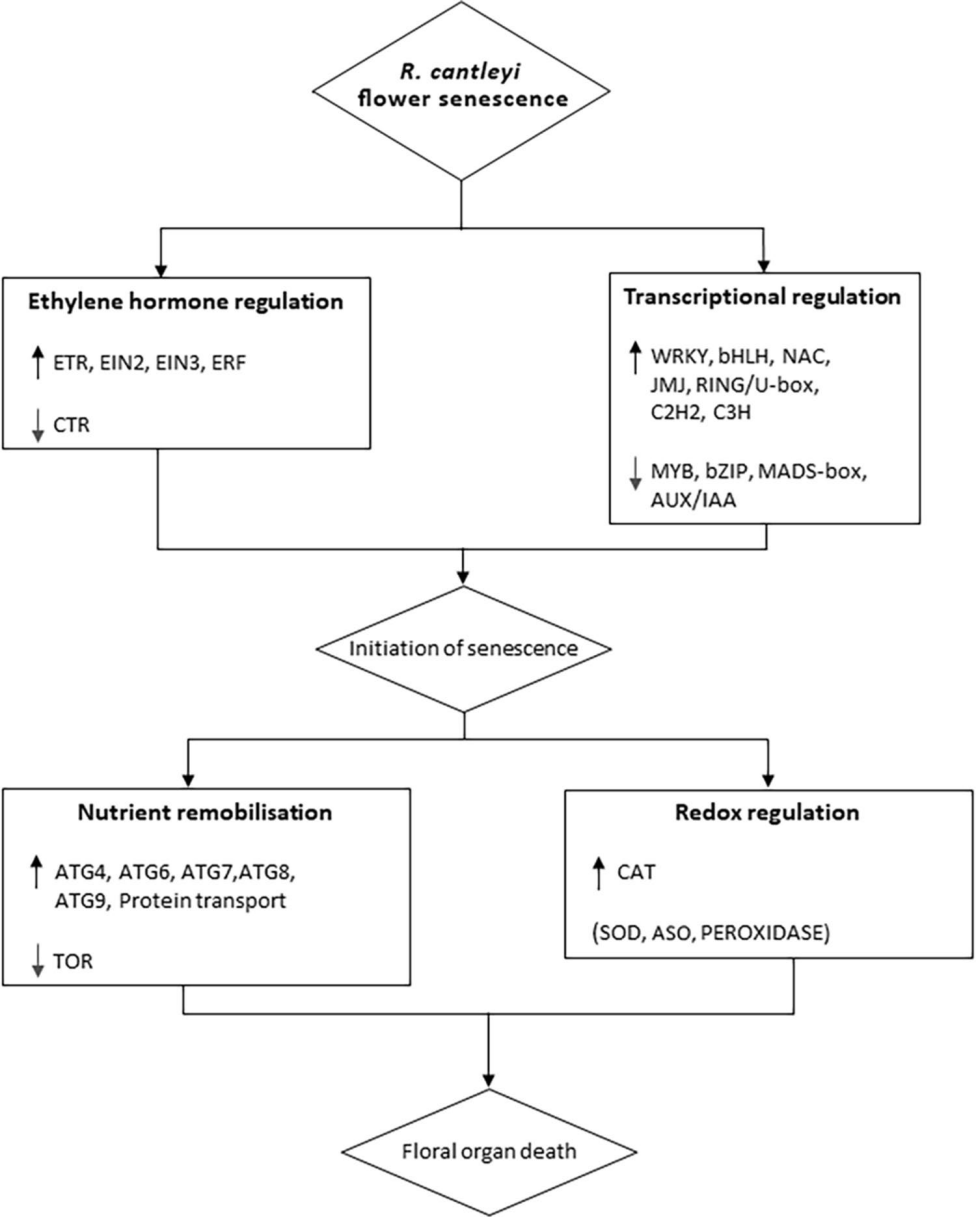
A model of flower senescence regulation in *Rafflesia cantleyi*. Upward arrows represent up-regulated genes and downward arrows represent down-regulated genes. Genes in parentheses are constitutively expressed. *ASO L-ASCORBATE OXIDASE*; *ATG AUTOPHAGY-RELATED GENES*; *CAT CATALASE*; *CTR CONSTITUTIVE TRIPLE RESPONSE*; *EIN ETHYLENE-INSENSITIVE*; *ERF ETHYLENE-RESPONSIVE TRANSCRIPTION FACTOR*; *ETR ETHYLENE RECEPTOR*; *SOD SUPEROXIDE DISMUTASE*; *TOR TARGET OF RAPAMYCIN*.

Results of the transcriptome data analysis allowed us to build a model for the regulation of senescence in *R. cantleyi*, based on a senescence regulation pathway proposed by Tripathi and Tuteja (2007)^81^ (Fig. 6). Senescence involves death at the cellular level known as PCD^13^. In *R. cantleyi*, PCD might be induced by environmental factors, such as biotic and abiotic stresses, which hasten the process of flower senescence and lead to the regulation of ethylene hormone and various transcription factors. Various pathways involving phytohormones and the regulation of transcription and signal transduction are known to activate the expression of senescence-associated genes^17^. The regulation of these transcription factors suggests activation of senescence-associated genes that are involved in the process of nutrient remobilization and redox regulation.

## Conclusion

Transcriptome generation and analysis focused on DEGs for two stages of *R. cantleyi* flowers yielded a model on the regulation of flower senescence in *R. cantleyi*. This model consists of the regulation of transcription factors and ethylene-related genes, together with nutrient remobilization, redox regulation, and the activation of senescence-associated genes, which ultimately lead to a coordinated death of a floral organ. The understanding of key genes and important pathways involved in *R. cantleyi* flower senescence will contribute towards further studies, including in the fields of biodiversity and conservation of this plant family.

## Methods

### Sample preparation and transcriptome sequencing

*R. cantleyi* flowers were collected from Bukit Lahar, Pahang, Malaysia, (Permission from the Pahang State Forestry Department—reference no. PHN. PHG. (PEM) 118/146 Bhg. 3 (123)). The collection of plant material complied with relevant regulations. Flower samples were obtained from two different stages of flower development, i.e., one day (flower stage 1; F1) and three days (flower stage 2; F2) after blooming (Fig. 1). Tissue samples were acquired from the perigone lobes of two different flowers that were collected from the same area of sampling and under a relatively similar environment. These samples were dissected and surface-sterilized using 10% (v/v) Clorox and rinsed with distilled water on site. Following that, the tissue samples were flash-frozen in liquid nitrogen before being transported to the laboratory. All the samples collected were stored at − 80 °C until further use. Total RNA extraction was carried out based on the modified CTAB method^82^. The extracted RNA was quantified using the ND-1000 Nanodrop spectrophotometer (Thermo Fisher Scientific Inc., USA). Before sequencing, the RNA integrity was quantified using the Agilent 2100 Bioanalyzer (Agilent Technologies, USA). cDNA libraries were constructed with the TruSeq® Stranded Total RNA Library Prep Kit (Illumina, USA) according to the manufacturer’s protocol. Subsequently, the libraries were sequenced using Illumina HiSeq™ 2000.

### Data pre-processing and *de novo* assembly of transcriptome

Raw sequencing reads generated (deposited to the NCBI SRA database with the accession numbers SRR7544088 (F1) and SRR7544087 (F2)) were quality-filtered using SolexaQA (v.2.2)^83^. High-quality reads were acquired by removing adaptor sequences and low-quality reads with Phred score less than 20 and read length less than 50 bp. Furthermore, the reads were screened for contaminating sequences from other organisms by aligning them using Bowtie2 (v2.3.0)^84^ to all genomes of bacteria (version 66, 7 July 2014), viruses, and fungi (version 73, 2 Nov 2015) from the NCBI database. Reads that aligned to these genomes were compared against the plant database Plaza 3.0^85^ and reads without any alignment to the plant genomes were discarded. Finally, the paired-end clean reads were subjected to* de novo* assembly using Trinity (version v2.2.0)^86^ and iAssembler (version v1.3.2)^87^. The *R. cantleyi* flower transcriptome assembly was deposited in the TSA database with the accession number GIQT00000000.

### Functional annotation of *R. cantleyi* transcriptome

To predict the putative functions of assembled unigenes, functional annotation was performed using BLASTX against NR and Swiss-Prot protein databases with an E-value cutoff of 1E-6. Unigenes involved in senescence were identified by performing similarity searches against 44 species of dicots and monocots in the Leaf Senescence Database (LSD 2.0)^88^. Gene ontology (GO) annotation was implemented on the annotated sequences from NR using the Blast2GO program and was further classified into three categories: biological process, molecular function, and cell component. The summary of GO classification was visualized using WEGO^89^. GO enrichment analysis was performed using Fisher’s exact test (FDR < 0.05) based on the up-regulated and down-regulated unigenes to predict the overrepresented GO function. KEGG pathway assignment was performed on unigenes using KEGG Automatic Annotation Server (KAAS)^90^ to predict metabolic pathways involved in the *R. cantleyi* flower.

### Differential gene expression analysis

Gene expression levels were estimated by mapping paired-end clean reads from each flower stage (F1 and F2) to the assembled transcriptome using the RSEM method^91^. Due to the great difficulties of on-site sampling of specific *Rafflesia* flower stages in the rainforest and the inability of cultivating *Rafflesia* for study under laboratory or controlled environment conditions, only one biological replicate was available for transcriptomic analysis. The gene expression was based on TPM (transcripts per kilobase million) that normalizes transcript length first before sequencing depth. Unigenes with TPM values greater than 0 were considered as expressed. By pairwise comparisons of the two libraries (F1 vs. F2), the DEGs were identified using the edgeR package^92^ based on the read counts of unigenes in different libraries. Trimmed Mean of M-values (TMM) normalization with an automatic method to estimate dispersion without replicates allowed single-replicate DEG analysis in edgeR, assuming that the counts are not too small. The significance of differential gene expression was judged by the false discovery rate (FDR ≤ 0.05) and fold change (|Log_2_FC|≥ 2) as the cut-off threshold.

### Gene expression analysis by RT-qPCR

Seven DEGs related to transcription factors, nutrient remobilization, redox regulation, and ethylene hormone pathway were selected from the transcriptome dataset and expression profiles were investigated by reverse transcription-quantitative polymerase chain reaction (RT-qPCR) analysis. Gene-specific primers (Supplementary Dataset S5) were designed using Primer-Blast. *TUBULIN* (*TUB*) and *ELONGATED FACTOR 1A* (*EF1A*) were selected as reference genes after they were identified as the most stable genes in the *R. cantleyi* flower by the Bestkeeper software^93^. For RT-qPCR analysis, 20 ng/µL DNAse-treated RNA was used as a template in a 20 µL total reaction with QuantiNova® SYBR® Green RT-PCR Kit (Qiagen, USA) according to the manufacturer’s instructions. The reactions were incubated in the CFX96™ Real-Time Detection System (Bio-Rad, USA) with the following cycling conditions: 10 min at 50 °C and 2 min at 95 °C for the reverse transcription, 40 cycles of 5 s at 95 °C and 10 s at 60 °C. In this analysis, three perigone lobes of each flower stage were treated as biological replicates, and each PCR reaction included three technical replicates. The changes in gene expression were calculated based on the 2^−∆∆Ct^ method^94^. Statistical analysis of the data was performed using unpaired Student’s *t*-test at a *P* < 0.05 significance level. Correlation analysis of relative expression values from RNA-seq and RT-qPCR was performed using Microsoft Excel.

## Supplementary Information

Supplementary Information 1.

Supplementary Information 2.

Supplementary Information 3.

Supplementary Information 4.

Supplementary Information 5.
